# Sirt6 deficiency contributes to mitochondrial fission and oxidative damage in podocytes via ROCK1‐Drp1 signalling pathway

**DOI:** 10.1111/cpr.13296

**Published:** 2022-07-17

**Authors:** Zhaowei Chen, Wei Liang, Jijia Hu, Zijing Zhu, Jun Feng, Yiqiong Ma, Qian Yang, Guohua Ding

**Affiliations:** ^1^ Division of Nephrology Renmin Hospital of Wuhan University Wuhan Hubei China; ^2^ Nephrology and Urology Research Institute of Wuhan University Wuhan Hubei China

## Abstract

**Objectives:**

Increasing evidence suggests that mitochondrial dysfunction is the key driver of angiotensin II (Ang II)‐induced kidney injury. This study was designed to investigate whether Sirtuin 6 (Sirt6) could affect Ang II‐induced mitochondrial damage and the potential mechanisms.

**Materials and Methods:**

Podocyte‐specific Sirt6 knockout mice were infused with Ang II and cultured podocytes were stimulated with Ang II to evaluate the effects of Sirt6 on mitochondrial structure and function in podocytes. Immunofluorescence staining was used to detect protein expression and mitochondrial morphology in vitro. Electron microscopy was used to assess mitochondrial morphology in mice. Western blotting was used to quantify protein expression.

**Results:**

Mitochondrial fission and decreased Sirt6 expression were observed in podocytes from Ang II‐infused mice. In Sirt6‐deficient mice, Ang II infusion induced increased apoptosis and mitochondrial fragmentation in podocytes than that in Ang II‐infused wild‐type mice. In cultured human podocytes, Sirt6 knockdown exacerbated Ang II‐induced mitochondrial fission, whereas Sirt6 overexpression ameliorated the Ang II‐induced changes in the balance between mitochondrial fusion and fission. Functional studies revealed that Sirt6 deficiency exacerbated mitochondrial fission by promoting dynamin‐related protein 1 (Drp1) phosphorylation. Furthermore, Sirt6 mediated Drp1 phosphorylation by promoting Rho‐associated coiled coil‐containing protein kinase 1 (ROCK1) expression.

**Conclusion:**

Our study has identified Sirt6 as a vital factor that protects against Ang II‐induced mitochondrial fission and apoptosis in podocytes via the ROCK1‐Drp1 signalling pathway.

## Graphical abstract

Schematic of the molecular action proposed in this study. Schematic depicting that Ang II‐induced sirt6 expression decline promotes mitochondrial fission and podocyte injury through ROCK1‐Drp1 signalling. SIRT6 reduction leads to increased levels of ROCK1, thereby enhancing Drp1 phosphorylation at the Ser637 site. The Drp1 phosphorylation finally results in podocyte injury by inducing mitochondrial fission and apoptosis.

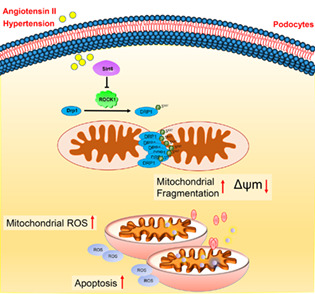

## 1 INTRODUCTION

The renin‐angiotensin‐aldosterone system (RAAS) plays a pivotal role in the occurrence and progression of chronic kidney diseases (CKD).^1^ Angiotensin II (Ang II) has been considered the primary effector of the RAAS.^2^ More than 20 years ago, it was concluded from indirect evidence that Ang II could induce mitochondrial reactive oxygen species (ROS) production.^3^ Our previous studies demonstrated that Ang II was able to directly induce podocyte damage.^4^, ^5^, ^6^, ^7^ However, the signalling molecules that mediate Ang II‐induced mitochondrial ROS production and dysfunction in podocyte injury remain to be defined.

Increasing numbers of studies suggest that the mitochondrial dynamics of fusion and fission are processes that maintain healthy mitochondria.^8^ Disorder of the fusion and fission dynamics in mitochondria is a pathological factor of acute kidney disease and CKD.^9^, ^10^, ^11^, ^12^, ^13^ Previous studies have shown that abnormal mitochondrial dynamics can induce abnormal ROS release from mitochondria and cell apoptosis. Several studies have indicated that Ang II can promote apoptosis through mitochondrial fission in a Drp1‐dependent manner.^14^, ^15^, ^16^

Emerging evidence suggests that epigenetic plays a key role in the occurrence and development of CKD.^17^ Sirtuins are best known for their roles in the regulation of multiple physical processes.^18^ Sirt6, a member of NAD^+^‐dependent enzymes, is located in mitochondria and plays an important role in regulating ageing, cancer, obesity and cellular energy metabolism.^19^ Sirt6 can control the transcription process of transcription factors by deacetylating histone H3. Several studies have revealed the essential role of Sirt6 in maintaining health. Male mice with higher levels of Sirt6 have extended lifespans.^20^ Conversely, Sirt6‐deficient mice exhibit accelerated functional decay, shortened lifespans, and progeroid syndrome as well as ageing, cancer and metabolic disorders.^21^

In previous studies, we showed that reduced expression of Sirt6 promoted cholesterol accumulation in Ang II‐treated podocytes.^22^ However, the role of Sirt6 in mitochondrial homeostasis in podocytes is still unknown. In this study, we confirmed that the inhibition of Sirt6 promoted Ang II‐induced podocyte injury by disrupting the homeostasis of mitochondrial dynamics.

## 2 METHODS

### 2.1 Animal models

Male C57BL/6J mice (8 weeks of age) were raised in a standard environment with regular humidity and temperature. The animal experimental proposal was reviewed and approved by the Animal Ethics Committee of Renmin Hospital of Wuhan University. The mice were infused with the normal saline or Ang II (700 ng/kg/min; Sigma‐Aldrich) via osmotic pumps for 28 days.^22^ After animal sacrifice, one‐quarter of one kidney tissue was fixed with 10% paraformaldehyde for paraffin preparation. A small piece of cortex tissue was preserved with 2.5% glutaraldehyde for electron microscopy as described previously.^5^ The remaining tissue was snap‐frozen in liquid nitrogen for subsequent analysis. Glomeruli were isolated from the opposite kidney as described previously^5^ and stored for Western immunoblotting. Podocyte‐specific Sirt6 knockout (KO) mice were obtained by crossing Sirt6‐floxed mice (C57BL/6J background; Cyagen Biosciences) with podocin‐Cre mice (C57BL/6J background; Jackson Laboratory). Sirt6^fl/fl^ littermates were used as controls. Both types of animals were infused with either normal saline or Ang II as described above. Urine and blood samples were regularly collected every 2 weeks before and after infusion pump implantation. In addition, 50 mg/kg of Mdivi‐1 was administered by intraperitoneal injection every other day for 28 days.

### 2.2 Morphological analysis

Kidneys were isolated and fixed using 4% paraformaldehyde for 30 min at room temperature. Kidney sections were stained with periodic acid silver methenamine. The ultrastructural changes in renal tissues fixed in 2.5% glutaraldehyde were assessed by electron microscopy as described previously.

### 2.3 Cell culture and treatment

Immortalized podocytes were kindly provided by Dr Moin A. Saleem and cultured as described previously.^6^ Briefly, cells were treated with Ang II (10^−7^ M) for 48 h and with vehicle (dimethyl sulfoxide) or Mdivi‐1 (5 μM).^6^

### 2.4 Western immunoblotting

IP/WB analysis was carried out as described previously.^6^ The antibodies against the following molecules were used for the analyses: Sirt6 (1:500; Abcam; #ab62739), Drp1 (1:1000; Abcam; #ab56788), mitofusin2 (Mfn2) (1:1000; Abcam; ab56889), mitochondrial fission protein 1 (Fis1) (1:1000; Genetex; GTX111010), p‐Drp1‐Ser616 (1:1000; Cell Signalling Technology; #3455), optic atrophy 1 (OPA1) (1:1000; ImmunoWay; YN2976), and p‐Drp1‐Ser637 (1:1000, Cell Signalling Technology; #4867).

### 2.5 siRNA and plasmid used for transfection

siRNA (QIAGEN) and the HiPerFect Transfection Reagent Handbook (QIAGEN) were used for cell transfection as previously described.^6^ The ROCK1 siRNA sequence was designed according to previous reports.^5^ For the overexpression of Sirt6, podocytes were transfected with a mixture containing 2 μg Sirt6 plasmids or pcDNA3.1 and 2 μl X‐tremeGENE HP DNA Transfection Reagent (Roche). Subsequently, cells were exposed to different conditions as indicated.^4^

### 2.6 Immunofluorescence assay and mitochondrial morphology assessment

Frozen sections were covered with blocking solution (10% bovine serum albumin), incubated for 30 min, fixed with 4% paraformaldehyde, and then incubated with primary antibodies. Then, the fluorescent secondary antibody was used to detect the primary antibodies according to a previously described experimental method.^5^ Mitochondrial morphology was assessed by staining the cells with MitoTracker Red (Invitrogen) as we previously described.^23^

### 2.7 ROS, ATP, mitochondrial membrane potential and apoptosis assay

Mitochondrial ROS levels were measured using MitoSOX Red (Invitrogen) as previously described.^24^ ROS levels in the glomeruli were measured using DHE (Sigma‐Aldrich) according to the instructions provided by the manufacturer. ΔΨm was measured using a commercial kit with JC‐1 (Beyotime), and ATP levels in the cell homogenates were measured using an ATP measurement kit (Beyotime). Cell apoptosis was evaluated by the apoptosis kit Annexin V‐FITC and 7‐aminoactinomycin D according to the method provided by the reagent supplier and as previously described.^23^

### 2.8 Statistical analyses

Quantitative data are expressed as the mean ± SEM, and SPSS 17.0 was used for relevant statistical analysis. Statistical comparisons of the groups were used with a one‐way analysis of variance, and the least significant difference test was used for multiple comparisons. Differences with *p* < 0.05 were considered statistically significant.

## 3 RESULTS

### 3.1 Mitochondrial morphology and Sirt6 expression in podocytes in Ang II‐infused mice

**FIGURE 1:**
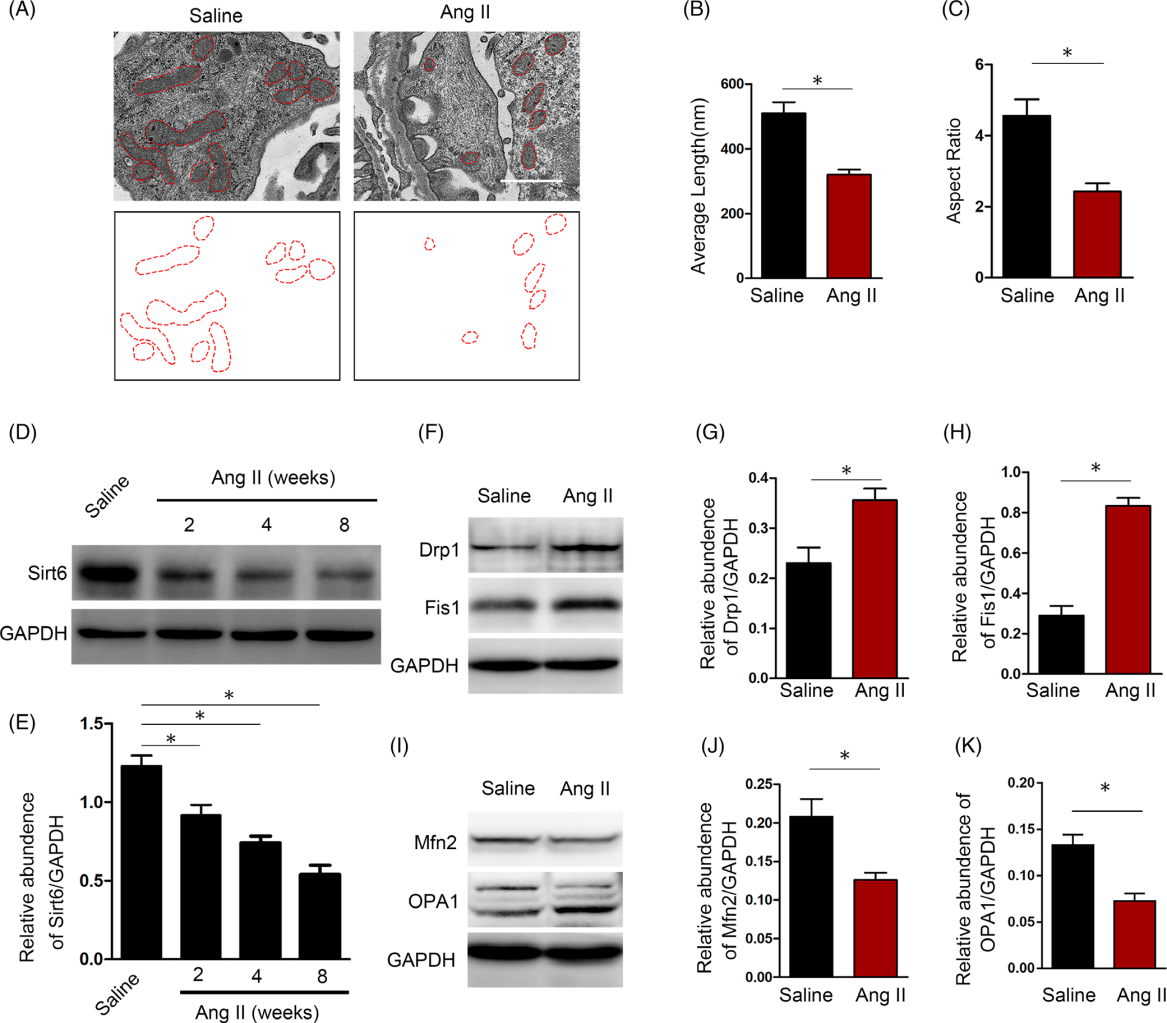
Ang II stimulation leads to mitochondrial fission and sirt6 downregulation in podocytes. (A) Electron microscopy ultrastructural image of podocyte mitochondria from the kidneys of normal or Ang II‐infused mice at 4 weeks. Scale bars = 1 μm. (B and C) Average length and aspect ratio of podocyte mitochondria from saline‐ and Ang II‐infused mice at 4 weeks. (D and E) Western blot of Sirt6 protein expression in glomeruli isolated at different time points of treatment and the expression of Sirt6 standardized to GAPDH. (F–H) Western blot of Drp1 and Fis1 protein expression in isolated glomeruli and the expression of Drp1 and Fis1 standardized to GAPDH. (I–K) Western blot of Mfn2 and OPA1 protein expression in isolated glomeruli and the expression of Mfn2 and OPA1 standardized to GAPDH. *p < 0.05, *n* = 5

To verify the effects of Ang II on mitochondrial dynamics in podocytes, an Ang II‐infused mouse model was established. Electron microscopy was used to evaluate the morphology of mitochondria in podocytes, and long rod‐shaped mitochondria were predominant in saline‐infused mice. However, shorter rod‐shaped mitochondria were found in podocytes from Ang II‐infused animals (Figure 1A). Mitochondrial morphology, in terms of both average length (Figure 1B) and aspect ratio (Figure 1C), in the podocytes from Ang II‐infused mice and saline‐infused mice was different. Moreover, compared to saline infusion, Ang II infusion profoundly reduced Sirt6 expression in the glomeruli from the early to late treatment time points (Figure 1D–E).

Since mitochondrial dynamics are regulated by a family of GTPases, several dynamin‐related GTPases that are involved in the fission and fusion processes of mitochondria were evaluated. Interestingly, the levels of proteins involved in the process of fission (Drp1 and Fis1) were dramatically upregulated in the glomeruli of Ang II‐infused mice compared with those of saline‐infused mice (Figure 1F–H). Conversely, the abundance of Mfn2 and OPA1, which are responsible for mitochondrial membrane fusion, was significantly decreased upon Ang II exposure (Figure 1I–K).

### 3.2 Podocyte‐specific deletion of Sirt6 aggravated mitochondrial fission and dysfunction in response to Ang II stimulation

**FIGURE 2:**
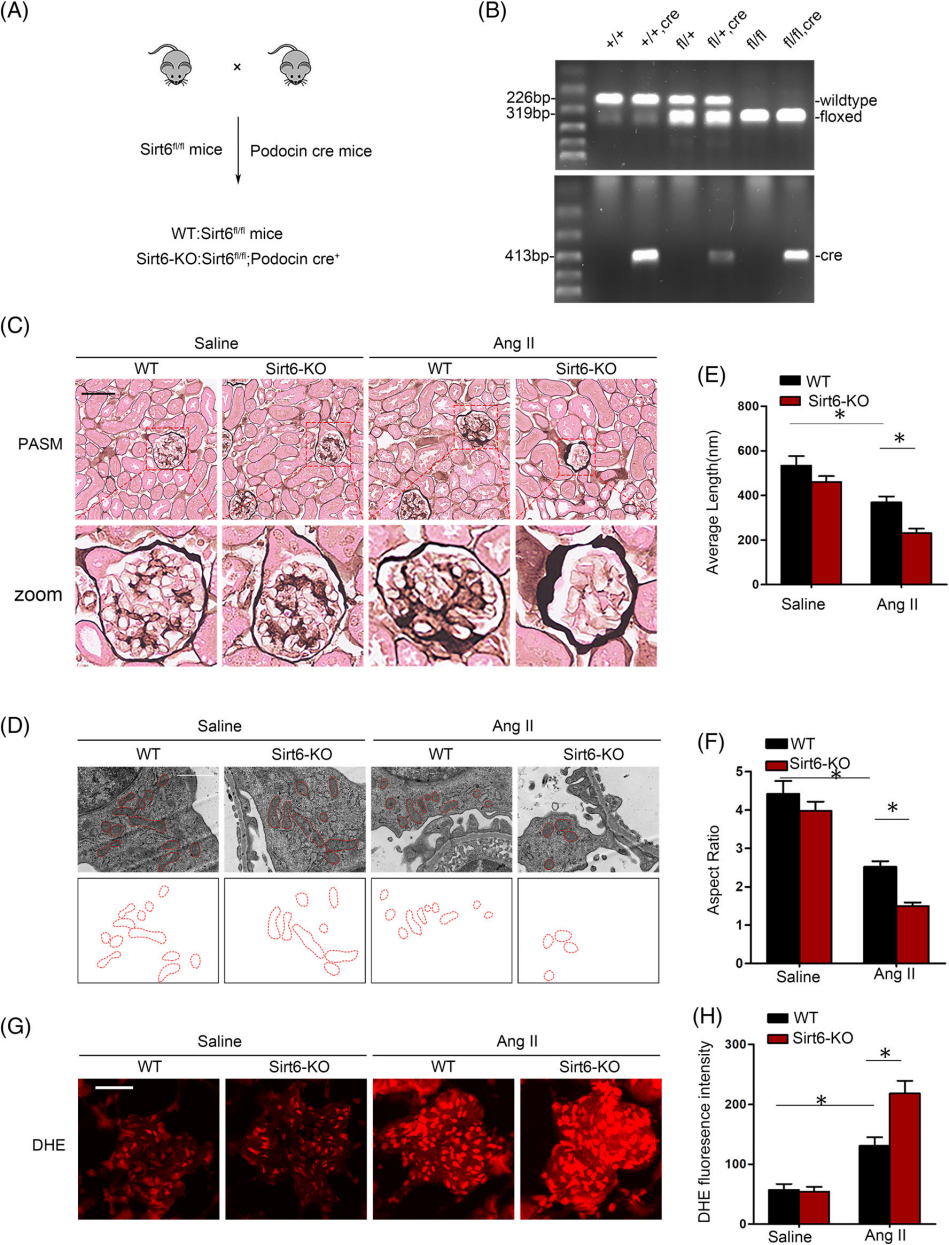
Sirt6 deficiency exacerbates mitochondrial changes in Ang II‐infused mice. (A) Schematic diagram illustrating the generation of podocyte‐specific Sirt6‐KO mice. (B) Representative pictures of PCR confirmation of mouse genotypes. (C) Representative micrographs of PASM staining of glomerular structure from the indicated groups. Bar = 50 μm. (D) Electron microscope ultrastructural image of podocyte mitochondria from WT and KO Ang II‐infused mice at 4 weeks. Scale bars = 1 μm. (E and F) Average length and aspect ratio of podocyte mitochondria from saline‐ and Ang II‐infused mice at 4 weeks. (G and H) The oxidative stress level was measured with dihydroethidium. Scale bar = 15 μm. *p < 0.05, *n* = 5

To further elucidate the effect of Sirt6 suppression in podocytes from Ang II‐infused mice, we generated podocyte‐specific Sirt6‐KO mice by intercrossing podocin‐cre mice with Sirt6^flox/flox^ mice. As recently reported, we confirmed the successful construction of Sirt6 conditional gene KO mice through Western blotting and immunofluorescence.^22^ The genotypes of the wild‐type (WT) and Sirt6 KO mice (Sirt6^fl/fl^, podocin‐cre, Sirt6‐KO) were further confirmed (Figure 2A,B). To further confirm the pathological changes that occur in mice after sirt6 gene KO, morphological abnormalities in the kidney were evaluated by light and electron microscopy. Ang II‐infused Sirt6‐KO mice showed more severe glomerular damage, including glomerular pyknosis, cellular proliferation and extracellular matrix accumulation, than Ang II‐infused WT littermate mice (Figure 2C). Notably, electron microscopy revealed more mitochondrial fission in Ang II‐infused Sirt6‐KO mice than in Ang II‐infused WT littermates (Figure 2D). As expected, the average length (Figure 2E) and aspect ratio (Figure 2F) of the mitochondria were significantly reduced in the podocytes from Ang II‐infused Sirt6‐KO mice, indicating that the loss of Sirt6 expression significantly exacerbated podocyte mitochondrial fission upon Ang II stimulation. In addition, ROS production was markedly increased in the glomeruli from Ang II‐infused Sirt6‐KO mice compared to those from Ang II‐infused WT littermates (Figure 2G,H). These observations suggest that Sirt6 deficiency contributes to increased mitochondrial fission and oxidative damage in podocytes.

### 3.3 Silencing Sirt6 expression promoted Ang II‐induced mitochondrial fission, oxidative damage and apoptosis in podocytes

**FIGURE 3:**
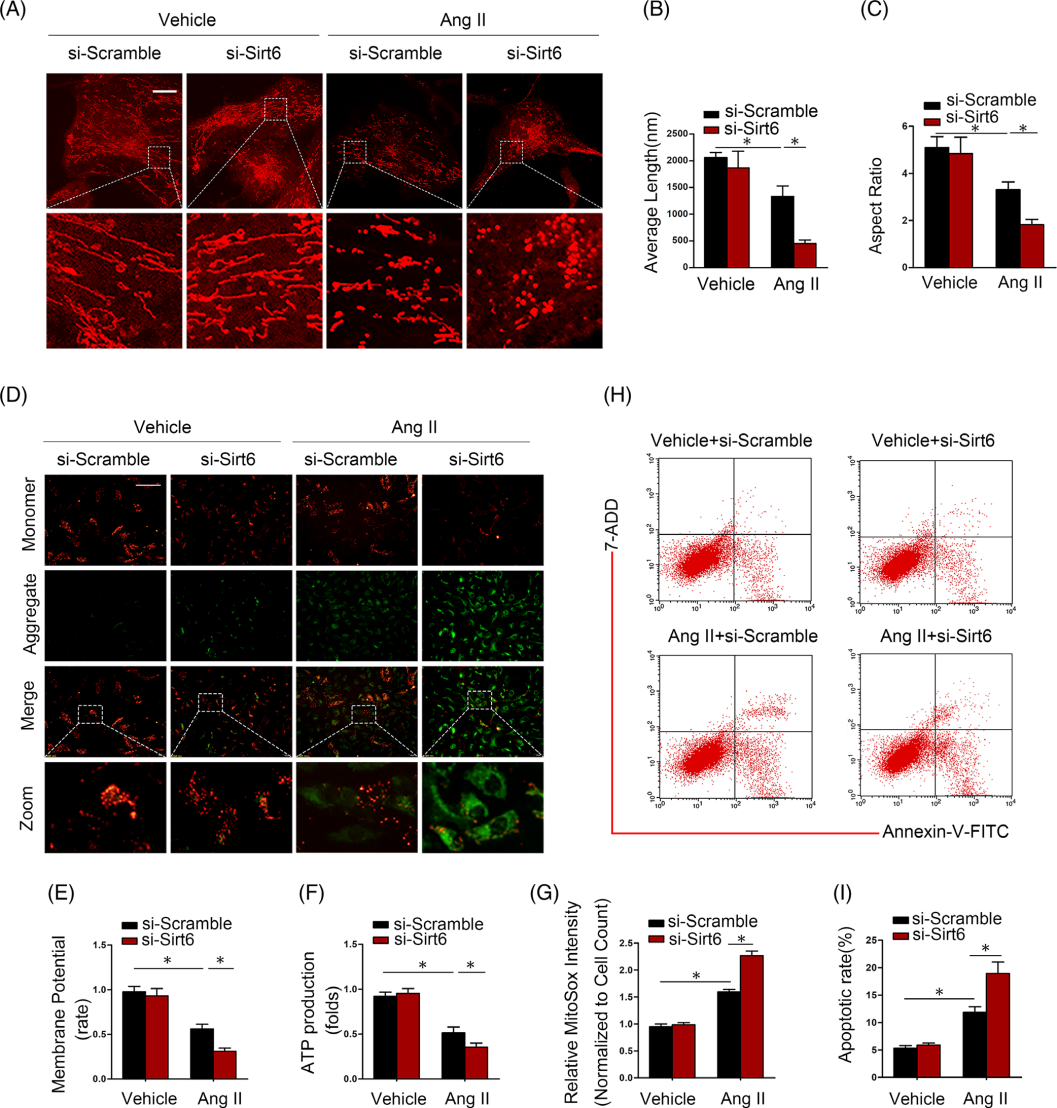
Silencing endogenous Sirt6 expression promotes Ang II‐induced mitochondrial injury and apoptosis in podocytes. (A) Representative image of mitochondrial morphology in podocytes stimulated with Ang II for 24 h as assessed by MitoTracker Red staining. Scale bar = 3 μm. (B and C) Average length and aspect ratio of podocyte mitochondria in each group. (D and E) The mitochondrial potential of podocytes under different conditions was detected via JC‐1 staining. Scale bar = 100 μm. (F) Comparison of ATP production levels in different groups. (G) Fluorescence intensity of MitoSOX Red staining of cells in different groups. (H and I) Analysis of cell apoptosis among different groups. *p < 0.05, *n* = 5

To explore the role of Sirt6 in vitro, Sirt6 siRNA was employed to knockdown Sirt6 expression as previously reported.^22^ To observe mitochondrial fission, a MitoTracker Deep Red immunofluorescence assay for mitochondria was carried out. We demonstrated that the mitochondria became punctate and fragmented upon Ang II exposure, and specific Sirt6 siRNA transfection exacerbated the changes in mitochondrial morphology in Ang II‐treated podocytes (Figure 3A). To quantify mitochondrial morphology, morphometric analyses were performed by calculating the average length and aspect ratio. Both parameters were significantly decreased in Sirt6 siRNA‐transfected podocytes under Ang II stimulation (Figure 3B,C). To further evaluate the mitochondrial function, we evaluated the mitochondrial membrane potential and APT production. Sirt6 knockdown exacerbated the Ang II‐induced mitochondrial membrane potential reduction (Figure 3D,E) and inhibited ATP production (Figure 3F). In addition, Sirt6 siRNA significantly exacerbated Ang II‐mediated mitochondrial ROS production (Figure 3G). Furthermore, Sirt6 siRNA significantly aggravated Ang II‐mediated podocytes apoptosis (Figure 3H,I).

### 3.4 Sirt6 overexpression in podocytes protected against Ang II‐induced mitochondrial dysfunction

**FIGURE 4:**
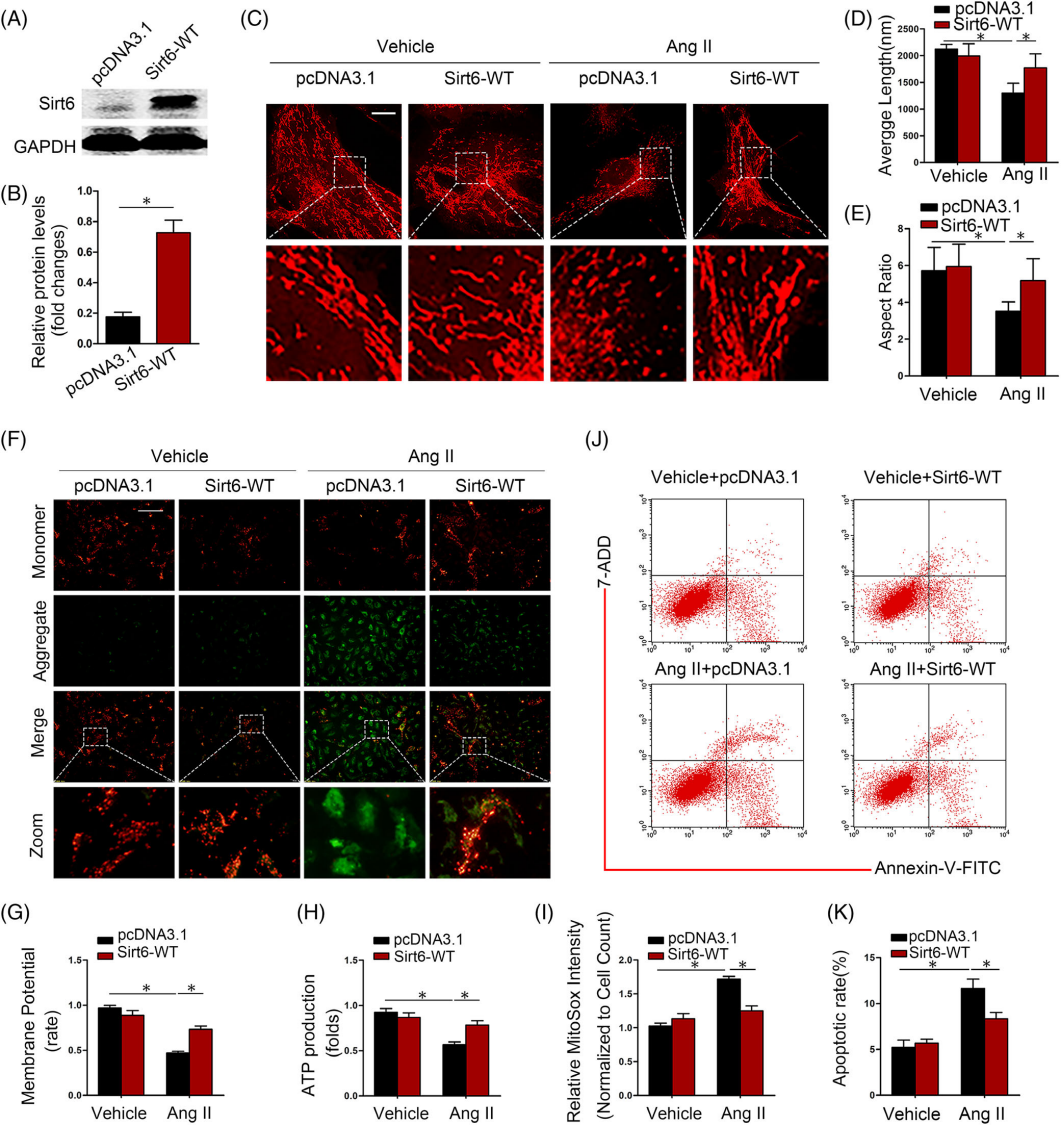
Sirt6 overexpression in podocytes protects mitochondria under Ang II stimulation conditions. (A and B) Assessment of transfection efficiency after pcDNA3.1 or Sirt6‐WT transfection. (C) Mitochondrial morphology assessed by MitoTracker Red staining of podocytes stimulated with Ang II for 24 h. Scale bar = 3 μm. (D and E) Average length and aspect ratio of podocyte mitochondria in each group. (F and G) The mitochondrial potential of podocytes under different conditions was measured via JC‐1 staining. Scale bar = 100 μm. (H) Comparison of ATP production levels in different groups. (I) Fluorescence intensity of MitoSOX Red staining of the cells in different groups. (J and K) Analysis of cell apoptosis among different groups. *p < 0.05, *n* = 5

To determine whether Sirt6 overexpression exerts protective effects on maintaining mitochondrial dynamics in podocytes after Ang II stimulation, a Sirt6 overexpression plasmid (Sirt6‐WT) was delivered to cultured podocytes. As expected, the protein expression of Sirt6 in Sirt6‐WT‐transfected podocytes was increased in comparison with that in vector‐transfected cells (Figure 4A,B). In Sirt6‐WT‐transfected podocytes, the Ang II‐induced fragmentation of the mitochondrial network was partially rescued (Figure 4C). With respect to the mitochondrial morphology index, the average length and aspect ratio were both significantly increased in Sirt6‐WT‐transfected podocytes treated with Ang II (Figure 4D,E). Moreover, Sirt6 overexpression prevented the Ang II‐induced decrease in mitochondrial membrane potential (Figure 4F,G) and ATP production (Figure 4H), Furthermore, the Ang II‐induced ROS accumulation in mitochondria was inhibited by Sirt6 overexpression (Figure 4I). Moreover, the high levels of apoptosis induced by Ang II were dramatically attenuated by Sirt6 overexpression (Figure 4J,K).

### 3.5 Sirt6 deficiency promoted mitochondrial fission by mediating the phosphorylation of Drp1 at the Ser637 site

**FIGURE 5:**
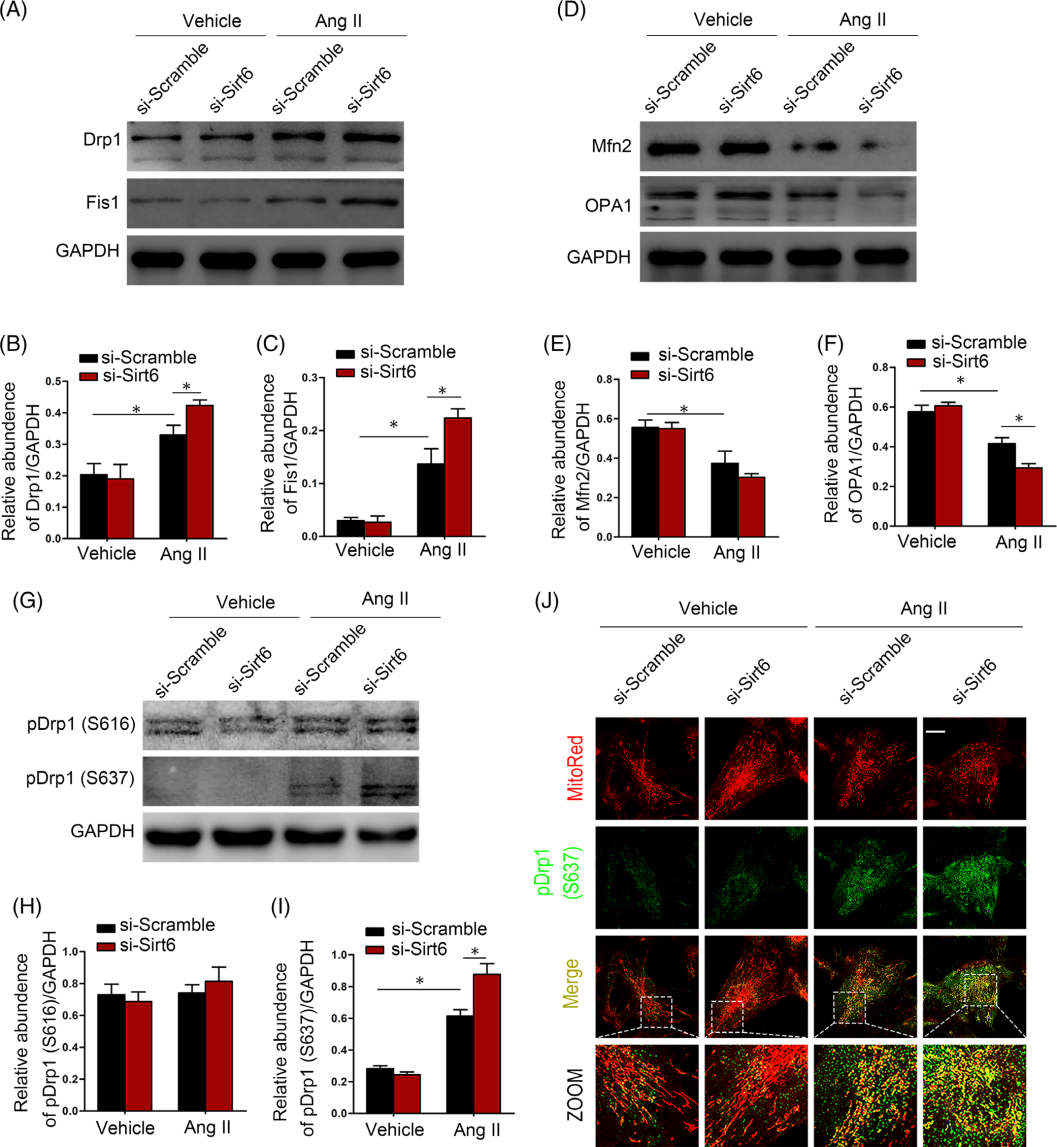
Sirt6 deficiency promoted mitochondrial fission by mediating the phosphorylation of Drp1 at Ser637. (A–C) Western blot of Drp1 and Fis1 protein expression in cultured human podocytes in different groups. (D–F) Western blot of Mfn2 and OPA1 protein expression in cultured human podocytes in different groups. (G–I) Western blotting was performed to analyse Drp1 phosphorylation levels among different groups. (J) MitoTracker and pDrp1(S637) costaining in podocytes cultured under different conditions in different groups. Scale bar = 3 μm. *p < 0.05, *n* = 3

To explore the precise molecular pathway by which Sirt6 participates in mitochondrial dynamics, we analysed Drp1 and Fis1 expression in cultured human podocytes. The results indicated that the expression of proteins involved in the process of mitochondrial fission (Drp1 and Fis1) was increased in Ang II‐treated podocytes (Figure 5A). In addition, the increased expression of Drp1 and Fis1 was exacerbated by Sirt6 knockdown only in the presence of Ang II (Figure 5B,C). In contrast to the proteins related to the fission process, the abundance of fusion proteins (Mfn2 and OPA1) was suppressed by treatment with Ang II, and this effect was exacerbated by Sirt6 knockdown (Figure 5D–F). It has been known that different phosphorylation patterns of serine residues in Drp1 result in different subcellular localizations of Drp1.^23^ The phosphorylation of Drp1 at two sites was increased (Figure 5G). Interestingly, only the Ser637 site of Drp1 underwent de novo phosphorylation after Ang II treatment. However, phosphorylation of Drp1 at Ser616 was not affected by Ang II treatment. Sirt6 deletion promoted Drp1 phosphorylation only in the presence of Ang II (Figure 5H,I). Consistent with the protein levels measured by Western blotting, the fluorescence intensity of pDrp1 (S637) was increased by Ang II stimulation, and this effect was enhanced with Sirt6 knockdown (Figure 5J). These results indicate that the Ser637 site of Drp1 is the target through which Sirt6 suppresses Ang II‐induced mitochondrial fission in podocytes.

### 3.6 The ROCK1‐Drp1 signalling pathway mediated Ang II‐induced mitochondrial fission

**FIGURE 6:**
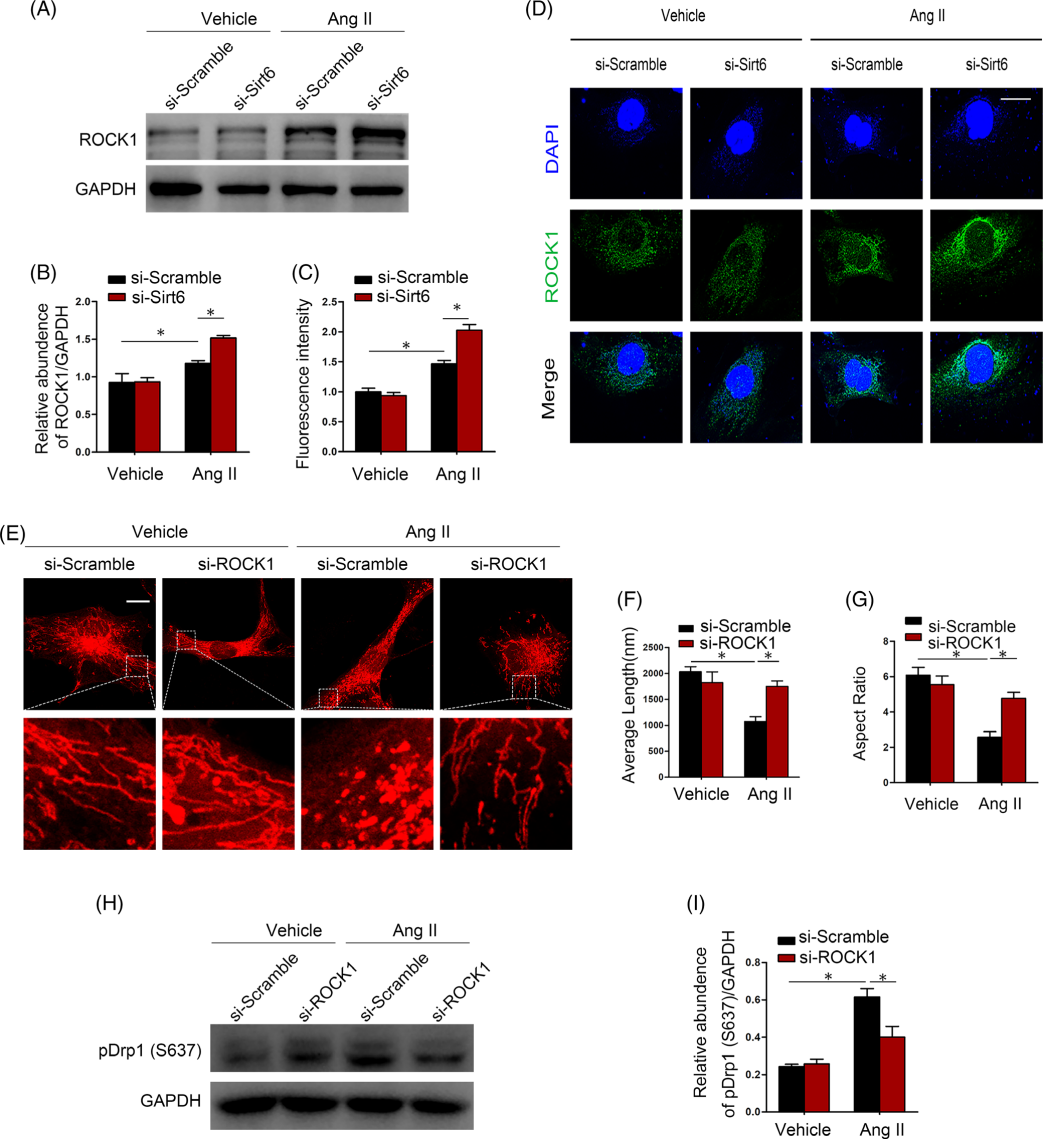
The ROCK1‐Drp1 signalling pathway mediates Ang II‐induced mitochondrial fission. (A and B) ROCK1 expression in human podocytes in different groups. (C and D) Immunofluorescence staining of human podocytes with DAPI and ROCK1 (green) in different groups. Scale bar = 20 μm. (E) Representative image of mitochondrial morphology in podocytes stimulated with Ang II for 24 h as measured by MitoTracker Red staining. Scale bar = 3 μm. (F and G) Average length and aspect ratio of podocyte mitochondria in each group. (H and I) Western blot of pDrp1(S637) protein expression in different groups. *p < 0.05, *n* = 3

Recent studies have shown that ROCK1 participates in the mitochondrial division by modulating the phosphorylation of Drp1.^25^ In addition to Drp1 phosphorylation, Sirt6 deletion augmented the Ang II‐induced increase in ROCK1 protein expression (Figure 6A,B). The changes in ROCK1 protein expression were further evaluated by immunofluorescence staining (Figure 6C,D). Conversely, ROCK1 siRNA rescued Ang II‐induced changes in mitochondrial morphology (Figure 6E), which was shown by assessing the morphometric indices of average length and aspect ratio (Figure 6F,G). Accordingly, Ang II‐promoted Drp1 phosphorylation at Ser637 was moderated by ROCK1 knockdown (Figure 6H,I). Altogether, our data indicated that the ROCK1‐Drp1 signalling pathway participated in Ang II‐induced mitochondrial fission.

### 3.7 Inhibition of Drp1 ameliorated Ang II‐induced mitochondrial injury in Sirt6‐deficient podocytes in vitro and in vivo

**FIGURE 7:**
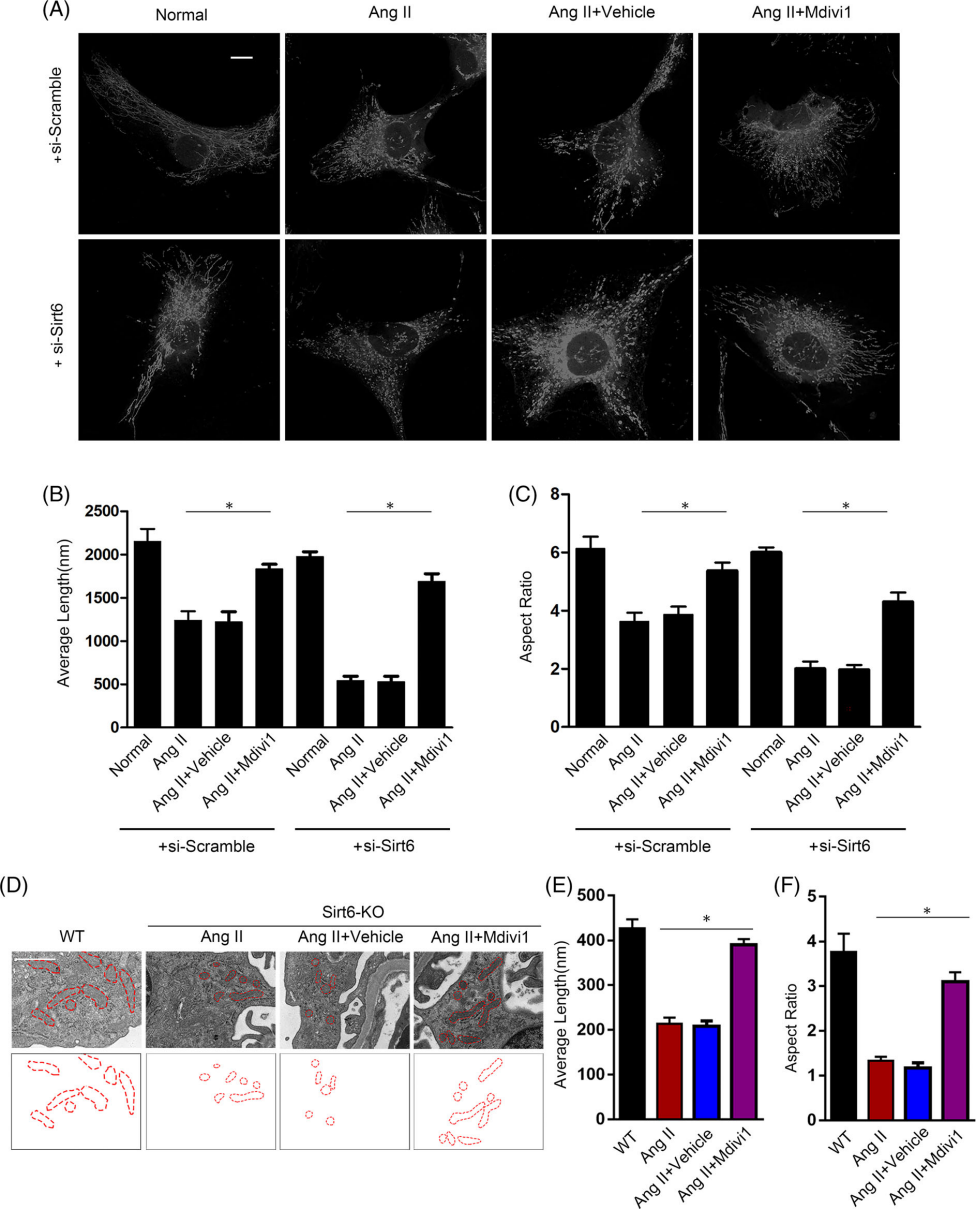
Mdivi‐1 alleviates mitochondrial dysregulation and apoptosis in vivo and in vitro. (A) Immunofluorescence staining images of mitochondrial morphology in Sirt6‐siRNA‐transfected podocytes treated with 20 μM Mdivi‐1 or vehicle (dimethyl sulfoxide [DMSO]) for 24 h. Scale bar = 3 μm. (B and C) Average length and aspect ratio of podocyte mitochondria in each group. (D) Electron microscopy ultrastructural image of podocyte mitochondria from the kidneys of the mice in the groups injected with Mdivi‐1 or vehicle (DMSO) every other day after Ang II administration. Scale bar = 1 μm. (E and F) Average length and aspect ratio of podocyte mitochondria from normal and Ang II‐infused mice at 4 weeks. *p < 0.05, *n* = 5

**FIGURE 8:**
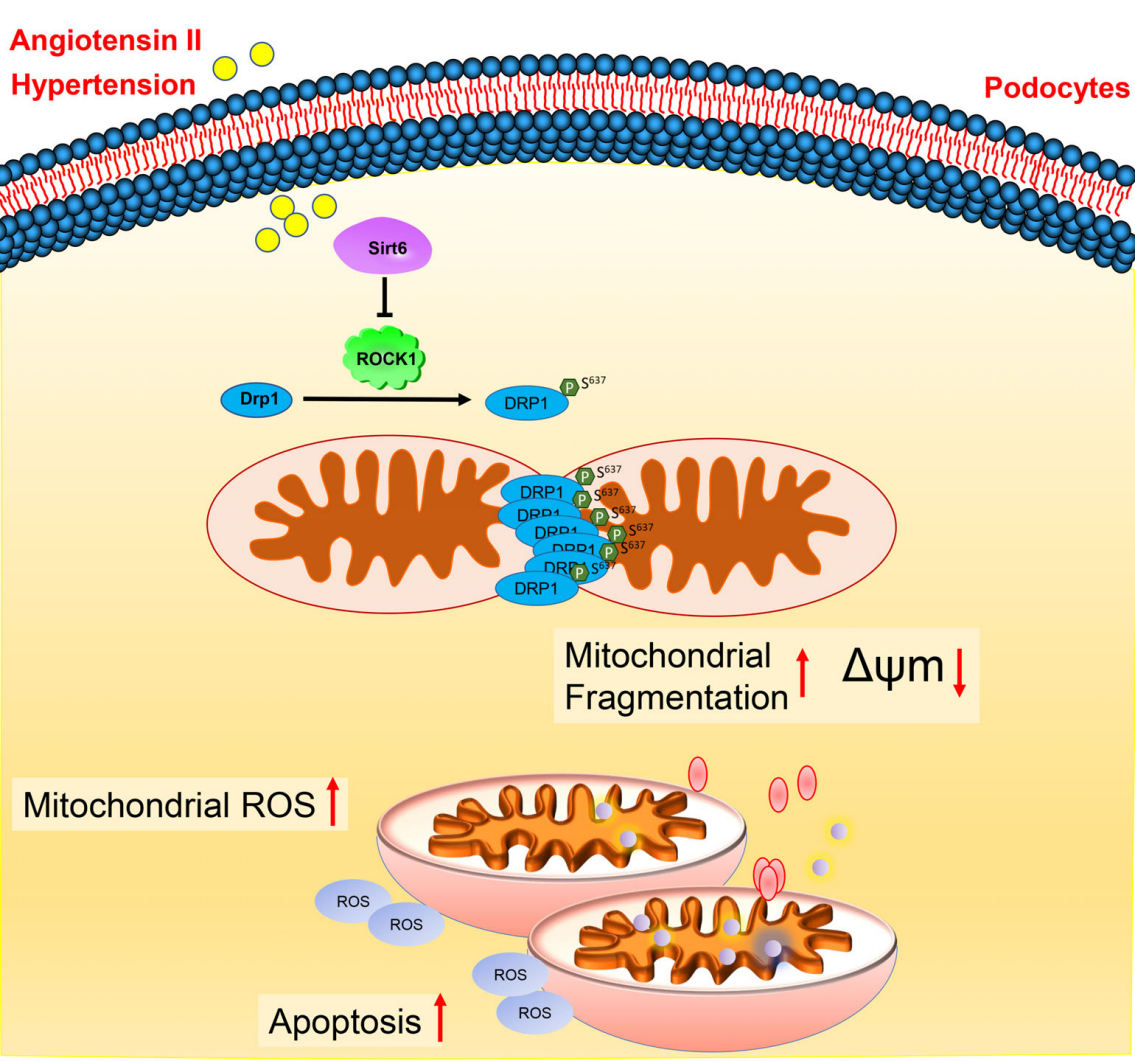
Schematic of the molecular action proposed in this study. Schematic of the molecular action proposed in this study. Schematic depicting that the decreased sirt6 expression caused by Ang II promotes mitochondrial fission and podocyte injury through ROCK1‐Drp1 signalling. Reduced SIRT6 levels lead to increased levels of ROCK1, thereby enhancing Drp1 phosphorylation at the Ser637 site. Drp1 phosphorylation ultimately results in podocyte injury by inducing mitochondrial fission and apoptosis

To test the direct effect of Sirt6 suppression on Drp1 translocation and mitochondrial fission, Mdivi‐1, which is an established pharmacological inhibitor of the GTPase activity of Drp1, was administered to Ang II‐treated podocytes. Interestingly, pretreatment with Mdivi‐1 dramatically attenuated the Ang II‐induced morphological changes even in the presence of sirt6‐specific siRNA, as evidenced by the average length and aspect ratio (Figure 7A–C). In addition, Mdivi‐1 significantly attenuated the Ang II‐induced decrease in mitochondrial potential and ATP generation (Figure S1A,B). Consistent with pretreatment with Mdivi‐1 in vitro, Mdivi‐1 was administered to Ang II‐infused mice on a Sirt6‐KO background. Electron micrographs indicated that Mdivi‐1 administration attenuated the mitochondrial fission observed in Ang II‐infused mice, as evidenced by the reduced average length and aspect ratio (Figure 7D–F). Therefore, Ang II stimulation may cause the downregulation of sirt6 and then activate the ROCK1‐Drp1 signalling pathway, which ultimately promotes mitochondrial fission and the resulting apoptosis in podocytes (Figure 8).

## 4 DISCUSSION

Activation of the RAAS has been implicated in the progression of CKD, including hypertensive renal injury, and inhibition of the RAAS partially delays the progression of proteinuria and end‐stage renal disease.^26^, ^27^ The oxidative stress‐induced damage caused by Ang II plays a critical role in pathological kidney damage, and previous studies have confirmed that oxidative stress and excessive ROS production are caused by an abnormal balance in mitochondrial dynamics.^28^, ^29^, ^30^, ^31^ In the present study, we found that Sirt6 expression was significantly suppressed in podocytes after long‐term exposure to exogenous Ang II. Furthermore, we confirmed that sirt6 deficiency promoted mitochondrial fission via the ROCK1‐mediated phosphorylation of Drp1 at the Ser637 site in vivo and in vitro. These findings indicate that Ang II promotes mitochondrial fragmentation and concurrent apoptosis in podocytes via the Sirt6‐ROCK1‐Drp1 pathway, which may offer a new therapeutic strategy for downstream overactivation of the RAAS in CKD.

Ang II is known as a key mediator in the progression of CKD, which affects actin cytoskeleton remodelling and induces kidney cell apoptosis, and in CKD.^6^, ^32^, ^33^, ^34^ The production of ROS can impact various pathophysiological processes. Excessive ROS production and enhanced redox signalling are associated with chronic diseases, including hypertension.^35^, ^36^ A large number of recent studies have confirmed that oxidative stress plays a pathologic role in hypertension‐related diseases. Oxidative stress is involved in multiple organ damage caused by hypertension, and hypertension itself can also promote oxidative stress.^37^, ^38^ However, the precise mechanism underlying Ang II‐induced ROS production in kidney disease is still unclear. Previous studies have confirmed that the increase in ROS production caused by Ang II stimulation is related to the excessive division of mitochondria,^39^, ^40^ but the key molecular mechanism linking Ang II activation with increased mitochondrial fission in the kidneys remains elusive. This study explored the role of Sirt6 in the pathogenesis of hypertension and focused on the role of mitochondrial fission in podocyte injury.

Accumulating studies show that the dynamic balance of mitochondrial fission and fusion plays an important pathophysiological role in a variety of diseases.^41^, ^42^, ^43^ The processes of mitochondrial fission and fusion are mainly regulated by dynamin‐related GTPases.^44^, ^45^ MFN1 and MFN2 are mainly responsible for controlling the fusion of the outer mitochondrial membrane. MFN1 can connect adjacent mitochondria through their HR2 domain. MFN2 is also related to the shuttling of Ca^2+^ between the endoplasmic reticulum and mitochondria. Fusion of the inner mitochondrial membrane is mainly coordinated by the OPA1 protein, which plays an important role in the maintenance of the mitochondrial crista structure. On the other hand, the mitochondrial division is mainly affected by Drp1 and Fis1.^46^ Drp1 molecules form structure between each other to shrink the mitochondrial membrane, while Fis1 anchors to the mitochondrial outer membrane and is related to the recruitment of Drp1. A number of studies have shown that Drp1‐mediated mitochondrial division is caused by a variety of stimulating factors. For example, hypoxia can trigger mitochondrial fission through Drp1 in cardiomyocytes.^47^ Recent studies reported that Ang II‐induced apoptosis by upregulating Drp1 expression in HUVECs.^40^ However, whether Drp1 is essential for Ang II‐induced podocyte injury and the upstream signals of Drp1 expression are still not fully understood. Our study showed that the upregulation of Drp1 expression was involved in the Ang II‐induced mitochondrial fission and concurrent apoptosis in podocytes. These results indicated that the apoptotic effect of Ang II might be caused by activation of the Drp1 signalling cascade and abnormal mitochondrial fission; In addition, Ang II‐induced mitochondrial fission was dependent on both ROCK1 activation and Drp1 phosphorylation. However, the mechanism underlying ROCK1 activation needs to be further explored.

Sirt6 is known to deacetylate the target transcription factor FoxO1. Forkhead box O transcription factors are involved in various signalling pathways, such as those related to stress resistance, metabolism, cell cycle arrest and apoptosis.^48^, ^49^ Accumulating evidence has indicated that FoxO1 also contributes to the progression of diabetes‐induced organ damage.^50^, ^51^ FoxO1 expression levels are increased in various tissues in diabetic mice and are involved in diabetes‐induced oxidative stress and cell apoptosis. FoxO1 can regulate the transcription of PDK4.^18^ Several recent studies demonstrated that FoxO1 directly mediated the transcription of a number of genes in podocytes under high glucose conditions.^52^, ^53^ Whether Sirt6 mediates Ang II‐induced apoptosis via ROCK1‐Drp1 signalling‐dependent mitochondrial dysfunction needs more study.

Sirt6 is a cytoprotective factor that participates in the regulation of cell metabolism and life span. Sirt6 could prevent mitochondrial defects and cell death via the NF‐κB pathway in hypoxia/reoxygenation‐induced myocardial injury. Moreover, overexpression of Sirt6 facilitated M2 macrophage polarization and ameliorated the progression of diabetic nephropathy.^54^ In addition, Sirt6 prevented the complications of pathological hypertension by maintaining endothelial function through the epigenetic regulation of Nkx3.2‐mediated GATA5 signalling.^55^ Our present study confirmed that Sirt6 deficiency exacerbated the Ang II‐induced disorder of mitochondrial dynamics and podocyte dysfunction via the ROCK1‐Drp1 signalling pathway. Sirt6 overexpression reversed Ang II‐induced mitochondrial fission and attenuated Ang II‐induced podocyte apoptosis. Furthermore, the application of a pharmacologic inhibitor of Drp1, namely, Mdivi‐1, significantly mitigated mitochondrial fragmentation and partially rescued Ang II‐induced pathological injury. In addition, recent studies have shown that Sirt6 participates in regulating the transcription factor GATA4 and protects against cardiomyocyte apoptosis via epigenetic activation.^56^

In summary, we have provided data demonstrating that Sirt6 suppression promotes mitochondrial fission by activating the ROCK1‐Drp1 signalling pathway, thus inducing kidney injury. This finding provides new insights into the regulatory mechanisms of mitochondrial dynamics under conditions of RAAS activation and offers new therapeutic approaches for podocyte injury.

## AUTHOR CONTRIBUTIONS

Briefly, Zhaowei Chen and Guohua Ding designed the study. Wei Liang, Jijia Hu, Zijing Zhu, Jun Feng, Yiqiong Ma and Qian Yang carried out experiments. Zhaowei Chen, Jijia Hu and Zijing Zhu analysed the data. Zhaowei Chen and Zijing Zhu produced the figures. Zhaowei Chen and Guohua Ding drafted and revised the paper. All authors approved the final version of the manuscript.

## CONFLICT OF INTEREST

The authors declare no conflict of interest.

## Supporting information


**Figure S1 A.** Quantification of JC‐1 fluorescence intensity among different groups as indicated. **B.** Quantification of intracellular ATP levels. Among different groups as indicated. *P < 0.05 compared with the normal group at the same time point.

## Data Availability

The data that support the findings of this study are available from the corresponding author upon reasonable request.
