# Supplementary material for: Sirt6 deficiency contributes to mitochondrial fission and oxidative damage in podocytes via ROCK1‐Drp1 signalling pathway
Source: Cell Prolif. 2022 Jul 17;55(10):e13296. doi: 10.1111/cpr.13296 (PMC9528772; doi:10.1111/cpr.13296)
Supplement: Supplementary file 1 — Figure S1 A. Quantification of JC‐1 fluorescence intensity among different groups as indicated. B. Quantification of intracellular ATP levels. Among different groups as indicated. *P < 0.05 compared with the normal group at the same time point. [file CPR-55-e13296-s001.docx]

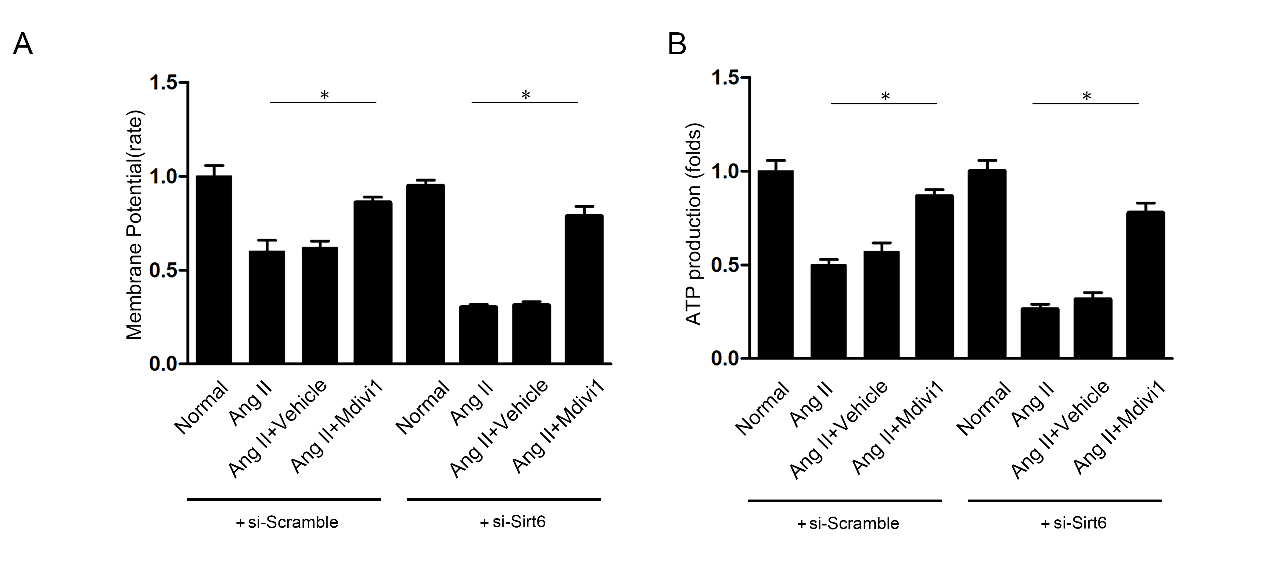


Supplementary Fig. S1. Mdivi1 attenuated the Ang II-induced decrease in mitochondrial potential and ATP generation. **A.** Quantification of JC-1 fluorescence intensity among different groups as indicated. **B.** Quantification of intracellular ATP levels. among different groups as indicated. **P* < .05 compared with the normal group at the same time point.
